# Terrell Medical Society

**Published:** 1889-06

**Authors:** 


### SOCIETY NOTES.

### TERRELL MEDICAL SOCIETY.

Dr. W. T. Strain in the chair. Dr. E. E. Smiley, secretary and treasurer. This society holds its regular meetings at Forney, Texas, on the first Mondays in each month, and at Terrell, Tex., on the third Mondays of each month.

Dr. Yates, of Poetry, produced a clinic of surgical interest: Man, aged 19, white, about two years ago, was kicked by a horse upon the anterior aspect of tibia, without fracturing the bone, merely causing a flesh wound which inflamed considerably, said inflammation gravitating to the lower third, invaded the periosteum, quickly and effectually producing necrosis at this point, but caused no pain for the period of two months. At this time the man was running, and of a sudden the limb failed him; was forced to stop work and use crutches. While holding the middle tibia firmly, the foot and lower third of tibia could be lifted latterly and up to an angle of 90° without exciting any pain.

Drs. Orr and Nelson favored immediate amputation, as likely to be followed with the best results, and cited a case rather similar, in which immediate amputation was followed by the most favorable results. Drs. Yates and Strain proceeded to amputate in about ten days and were surprised to find the periosteum and muscular structure entirely diseased from the lower third to the knee joint, and finally concluded to disarticulate at the knee joint; which being done, resulted favorably. The bones were presented before our society for inspection, and there was an artificial joint being formed in the lower third which accounts for the limb being movable in a latteral direction.

Dr. Fry, of Elmo, reported a case of jaundice. The matter as vomited presented the appearance of the color of gold; patient had chills at short intervals; bowels became deranged; the brain became implicated; patient became comatose and delirious, which finally ceased; severe tenderness of abdomen; tongue heavily coated; conjunctiva very much jaundiced; urine scanty and of high sp. gr. Gave calomel in small doses at short intervals; quinine, digitalis and nitro-muriatic acid. Patient made a good recovery.

Dr. Garrett reported a similar case, passing much bloody urine, and at the suggestion of Dr. L. M. Stroud, of Forney, used digitalis, the hot bath, bic. of pot., and gave quinine. Urine soon cleared; bowels were soon regulated and patient made a good recovery.

Dr. L. M. Stroud antagonized the use of quinine in these intensely malarial diseases, agreeing with the best authority that its tendency is to excite hemorrhage, and this hemorrhage is in all probability poured into the body of the kidney, hence the great and unlooked for trouble found existing in the kidneys. The views, as advanced by Dr. Stroud, are the result of mature deliberations. You will find, by referring to the fifth volume of Sajou's Universal Medical Compendium, embracing all views worthy of being practically applied, the mode in which Dr. Stroud uses chloroform and such agents. Dr. Stroud, though quite unassuming and retiring in his disposition, is ere long to appear as standard authority on medicine and surgery.

Dr. Jones stated that he had received very satisfactory results from quinine, buchu and pot. acetas in cases in which the urine was very bloody and inclined to be foamy, causing much straining and indescribable agony.

Drs. Orr and Fry regards the bloody condition of the urine in jaundice as due to destructive blood corpuscles and that the system thus seeks to eliminate this which now becomes a materies morbi in the body of the kidneys, making its exit through the ureters and bladder.

Dr. Orr regards jaundice in certain conditions as quite similar to yellow fever and swamp fever.

Dr. White read an able and highly interesting paper on hysteria, stating that this affection possesses various avenues of approach. In some peculiar cases it points to mental aberration. Having ample opportunities for practical observation, his views were eminently appreciated.

Dr. Stroud held that in jaundice, because of the inactivity of the liver, the skin being called upon to aid, eliminated the bile pigment, hence the color of the skin in jaundice. The doctor also stated that hysteria frequently appeared in rather aggravated forms, whith were to be ascribed to diseased or dislocated uterus or its appendages.

Dr. Fry coincided with the views that had been advanced concerning the difficulty of tracing hysteria to its real cause and referred to the silver hair among the gray as the result of such investigating efforts.

Dr. Wallace had had an extensive experience with jaundice, and that in typical cases the prognosis was unfavorable.

Dr. Inabnit read a very interesting paper upon the subject of "Labor among the primitive people of all races and their various modes of proceedure, compared to the present." Great honor and credit certainly are due to the efforts of investigation.

Dr. Wallace then read an eloquent paper upon the subject "Medical Youth, Manhood, Age." [Paper was published in the April number of DANIEL'S TEXAS MEDICAL JOURNAL, 1889.]

Dr. Garrett reported a case which he had diagnosed Milliary Tuberculosis. Female, aged 35; first attacked with chills and fever; cough, which has continued every day since he was first called to see the case. Night sweats set up; cough more obstinate; bowels constipated; is very much emaciated; spleen enlarged; also the liver; temperature for the most time is 102°, pulse rate, 120; respiration, 28.

Dr. White was of the opinion that milliary tuberculosis often exists in a latent state and is not detected till some time elapses.

Dr. Stroud endorsed these views, since night sweats associated with emaciation, should excite our suspicions concerning the lungs, and stated that he had seen similar cases in the dead house, in which the lungs were full of tuberculous infiltration, when such was not supposed to exist.

Dr. Stroud, in speaking of cases with compromised respiration, reported a case of asthma which was relieved as if by the magician's power, by one 40 grain dose of antipyrine, it not being necessary to repeat the dose.

Dr. White reported, that there had been some 18 or 20 cases of erysipelas at the North Texas Insane Asylum and that he had received splendid results from pilocarpine, used hypodermically, which seemed to arrest the onward march; free diaphoresis would result, and that one dose was generally sufficient, and then followed with iron mur. tinc. and quinine.

Dr. Stroud explained the physiological action of pilocarpine as exciting the amœboid movement of the white corpuscles in increasing the capillary capacity.

Dr. Monday reported several cases of erysipelas, treated as above with good results.

Dr. White took the ground that pilocarpine primarily affected the sudoriferous glands and indirectly affected the capillaries.

Dr. Jones reported a case of pneumonia, associated with mumps.

Dr. Stroud cited one peculiarity in reference to parotitis, viz.: That the temperature is always greater in the mouth than that taken in the axilla, and from this fact he never takes the temperature in a case suspected of mumps; but in doubtful cases arrives at a correct diagnosis by comparing these temperatures.

---

Dr. W. P. Fleming, of Georgetown, has been appointed Chief Surgeon of the Columbia & Puget Sound Railroad, with headquarters at Seattle, Washington, Territory.

